# Potential Pharmacokinetic Drug-Drug Interactions between Cannabinoids and Drugs Used for Chronic Pain

**DOI:** 10.1155/2020/3902740

**Published:** 2020-08-13

**Authors:** Marta Vázquez, Natalia Guevara, Cecilia Maldonado, Paulo Cáceres Guido, Paula Schaiquevich

**Affiliations:** ^1^Departamento de Ciencias Farmacéuticas, Facultad de Química, Universidad de la República, Montevideo, Uruguay; ^2^Unidad de Farmacocinética Clínica, Farmacia, Hospital de Pediatría JP Garrahan, Buenos Aires, Argentina; ^3^Medicina de Precisión, Hospital de Pediatría JP Garrahan, Buenos Aires, Argentina; ^4^Consejo Nacional de Investigaciones Científicas y Técnicas, Buenos Aires, Argentina

## Abstract

Choosing an appropriate treatment for chronic pain remains problematic, and despite the available medication for its treatment, still, many patients complain about pain and appeal to the use of cannabis derivatives for pain control. However, few data have been provided to clinicians about the pharmacokinetic drug-drug interactions of cannabinoids with other concomitant administered medications. Therefore, the aim of this brief review is to assess the interactions between cannabinoids and pain medication through drug transporters (ATP-binding cassette superfamily members) and/or metabolizing enzymes (cytochromes P450 and glucuronyl transferases).

## 1. Introduction

A drug-drug interaction (DDI) occurs when one drug alters the clinical effect of another. Drug interactions occur on pharmacodynamic and pharmacokinetic levels. In the first case, one drug may alter the sensitivity or responsiveness to another drug. Pharmacokinetic DDIs occur when a drug alters the absorption or disposition (distribution and elimination) of a concomitantly administered drug. This change can lead to an altered quantity of drug at the site of action affecting the magnitude and duration of the effect. In this scenario, a drug is a perpetrator referring to the one that causes an effect on the substrate drug, for example, by inducing or inhibiting drug-metabolizing enzymes. Although DDIs are often associated with toxicity or therapeutic failure [1], sometimes they can produce beneficial effects to the patient (i.e., improving the bioavailability of a drug and producing additive or synergistic effects) [2]. In any case, clinicians must be familiar with DDIs in order to improve prescribing tools.

During the last 5 years, a dramatic rise in the use of cannabis led to an increased number of patients taking it simultaneously with their previous medication. This situation could result in several problems as cannabinoids may be classified as either perpetrators or substrates depending on the concomitant drugs leading to altered exposure, adverse events, and/or lack of clinical efficacy. However, scarce evidence is available about cannabis drug interactions with potential implications in clinical efficacy and safety.

The endocannabinoid system has been recognized as a potential therapeutic target. Either highly purified cannabidiol (such as Epidiolex recently approved in the United States for use in Lennox–Gastaut or Dravet syndrome) or formulations with different *Δ*^9^-tetrahydrocannabinol (THC) to cannabidiol (CBD) ratios (such as Sativex, an oromucosal spray for the treatment of multiple sclerosis-associated spasticity) are being investigated for other disease states. Although the use of cannabinoids for the treatment of pain is supported by some controlled clinical trials [3–5], currently and according to systematic reviews and meta-analysis [6–8], there is only moderate evidence to support the use of cannabinoids in treating chronic pain and larger and higher quality clinical trials are needed. Despite this fact, chronic pain relief is by far the most common condition cited by patients using cannabis for medical purposes and very little is known about potential pharmacokinetic interactions with common medication prescribed for chronic pain.

Nowadays, even cannabinol (CBN), a byproduct of THC degradation, is being studied for its analgesic effect [9].

CBD, THC, and CBN are extensively metabolized in the liver and in the intestine. Mainly CYP2C19 and, to a lesser extent, CYP3A4 are implicated in CBD biotransformation [10, 11]. CBD can also undergo direct conjugations via UDP-glucuronosyltransferase (UGT) enzymes, such as UGT1A9, UGT2B7, and UGT2B17 [12, 13]. THC biotransformation is primarily dependent on CYP2C9 and CYP3A4 isoenzymes [14], but UGT enzymes play a critical role in metabolizing THC metabolites (THC-OH, THC-COOH) as well [12]. CBN is metabolized by CYP2C9 and CYP3A4 and can also undergo direct glucuronidation by hepatic UGT1A9 and the extrahepatic UGT1A7, UGT1A8, and UGT1A10 [12, 14].

CBD is not only a substrate but also an inhibitor of CYP450 enzymes and UGTs. In addition, some isoenzymes of the cytochrome P450 system or UGTs are also subjected to inhibition by THC and CBN [11, 15–25].

Regarding the inducing activity of cannabinoids, smoked cannabis may increase the clearance of drugs metabolized by CYP1A2 [24, 25], resulting in lower concentrations of these drugs and perhaps in therapeutic failure.

Furthermore, *in vitro* and animal studies have shown that CBD, THC, and CBN interact in some way with ATP-binding cassette superfamily: breast cancer-resistant protein (Bcrp) and glycoprotein P (Pgp). Thus, a significant impact on the absorption and disposition of other coadministered drugs that are also substrates of these transporters may be expected. According to some preclinical studies [26–29], CBD inhibits Pgp and Bcrp. Even though inhibitors are often substrates, different *in vitro* and animal studies show that CBD is not a Pgp substrate [30, 31] and it acts provoking a downregulation in Pgp expression. THC and CBN could also deregulate Pgp, Bcrp, and multidrug-resistant protein (MRP) 1-4 expression [15]. An overview of the effect of cannabinoids on CYP450 isoenzymes, UGTs, and efflux transporters is summarized in Table 1.

As cannabinoids are often used as add-on therapy, the occurrence of DDIs seems more plausible. Therefore, their use in the therapy could interfere with the disposition of other drugs that undergo the same metabolic pathways. Nonetheless, few studies in humans have been carried out and reported in the literature about DDIs of cannabinoids with other prescribed medications [32–34] and some of them are only case reports [35–37].

Although *in vitro* or animal studies about DDIs should not be extrapolated to human beings, healthcare providers should be aware of clinically important DDIs leading in some cases to therapeutic improvement or in other cases to therapeutic failure or toxicity. Therefore, this review addresses a comprehensive overview of potential pharmacokinetic interactions affecting drug metabolism enzymes such as cytochrome P450 or UGTs and membrane efflux transporters between cannabinoids and drugs used for chronic pain.

## 2. Methodology

Electronic databases of published scientific literature were the main source for this review. The *in vitro* and *in vivo* research findings and clinical case reports were searched from PubMed, Google Scholar, and Cochrane Library. Some studies were identified with Google search. Additional articles of interest were obtained through cross-referencing of published literature. The primary key terms used were “pharmacokinetics,” “drug interactions,” “cannabinoids,” “metabolizing enzymes,” “efflux transporters,” and “chronic pain medication.” Only English language papers were taken into consideration.

## 3. Drug-Drug Interactions

### 3.1. Cannabinoids-Opioids

The conventional opioids most commonly used for chronic pain management are morphine, oxycodone, codeine, methadone, tramadol, and fentanyl. Most opioids exert an analgesic effect through binding to the *μ* opioid receptor except for tramadol and methadone that include both opioid and nonopioid components [38, 39].

Morphine is glucuronidated via UGT2B7 to morphine-3-glucuronide (M3G) and morphine-6-glucuronide (M6G), being the latter a highly active analgesic [40].

Oxycodone is metabolized in the liver by CYP3A4/5 and CYP2D6. An active metabolite (oxymorphone) is formed by CYP2D6 [41, 42]. Oxycodone glucuronidation is carried out by UGT2B7 and UGT2B4 while oxymorphone is glucuronidated mostly by UGT2B7 [43].

CBD inhibits UGT2B7, and thus, a lower M6G to morphine ratio should be expected and less analgesic potency. Moreover, CBD, THC, and CBN inhibit CYP2D6 affecting oxymorphone formation and thus reducing analgesic effect. Therefore, if the interactions mentioned above take place, perhaps less analgesia would be seen with the combination of cannabis and these two opioids. However, several studies in the literature report that cannabis enhances the analgesic effects of opioids, thereby allowing for lower doses [44–47]. Furthermore, Abrams et al. [48] found that vaporized cannabis given to patients with chronic pain on opioid therapy (morphine or oxycodone) increased the analgesic effect of opioids but no significant differences were observed in the mean plasma concentration-time curves for morphine and oxycodone with and without cannabis treatment. These authors suggested pharmacodynamic interactions between opioids and cannabinoids. However, as opioid delivery to the brain is influenced by ATP-binding cassette transporters [49–51], a pharmacokinetic interaction should not be neglected.

Several cytochrome P450 enzymes are involved in methadone metabolism: CYP3A4, CYP2B6, and CYP2C19 and, to a lesser extent, CYP2C9, CYP2C8, and CYP2D6. It has become clear nowadays that CYP2B6, rather than CYP3A4, is the predominant P450 responsible for clinical methadone disposition [52]. CBD is a strong inhibitor of CYP2B6, so increased levels of this opioid and a greater analgesic potency might be observed. An increased plasma level of methadone was observed in a pediatric patient receiving CBD [36], which decreased fourteen days after CBD was discontinued.

Some authors [53] concluded that morphine and methadone analgesia was greater in mice lacking Pgp. Hassan et al. [54] found that oxycodone is a Pgp substrate *in vivo*. Some studies [50, 55, 56] suggested that this efflux transporter limits the entry of some opiates into the brain and that administration of Pgp inhibitors or drugs that downregulate Pgp expression can increase the sensitivity to these opiates. This fact, rather than enzyme inhibition by cannabinoids, could be the explanation of augmented analgesic potency of opiates, and the need of lowering their doses in the presence of cannabinoids as efflux transporters are deregulated by cannabinoids. This could be the case for morphine, oxycodone, and methadone as they are substrates of efflux transporters.

Neither codeine nor tramadol is Pgp substrates [51, 57]. The polymorphic CYP2D6 regulates the O-demethylation of codeine and tramadol to more potent metabolites: morphine and O-desmethyl-tramadol, respectively. Tramadol undergoes another metabolic pathway catalyzed by CYP3A4 and CYP2B6.

According to some authors [58], if the subject is a poor metabolizer, inadequate analgesia can be observed. If CBD, THC, or CBN inhibition of CYP2D6 predominates, the analgesic effects of tramadol and codeine will be reduced. However, the fate of the active metabolites has to be taken into account as well. O-desmethyl-tramadol undergoes inactivation by UGT2B7 and UGT1A8 [59], and morphine as stated before is a Pgp substrate. If cannabinoids interfere in the elimination of these metabolites either by inhibiting UGT2B7 or by deregulating efflux transporter expression, the result will be the opposite. Further studies are necessary in order to assess cannabinoid influence on codeine and tramadol.

Fentanyl is mainly metabolized by CYP3A4 and is a Pgp substrate [50, 60]. Although some authors found no interaction between fentanyl given intravenously and CBD [61], plasma levels of fentanyl were undetectable before and after the administration of CBD. Therefore, deeper research is necessary in order to conclude on a possible pharmacokinetic interaction.

To sum up, if opioids and/or their active metabolite levels are increased when taken along with cannabinoids, an enhanced analgesic activity can be observed.

### 3.2. Cannabinoids-Acetaminophen

Acetaminophen (paracetamol) is a drug with analgesic and antipyretic properties widely used for pain relief. Although its analgesic effect is weaker in comparison with nonsteroidal anti-inflammatory drugs (NSAIDs), it can be considered as a first-line option among nonopioids due to a more favorable safety profile. However, high concentrations can induce liver damage, and therefore, daily doses should not exceed 4 g [62].

Acetaminophen glucuronidation by UGT1A1, UGT1A6, UGT1A9, and UGT2B15 is the main biotransformation pathway, and only a minor fraction of the drug is oxidized to the highly reactive metabolite N-acetyl-p-benzoquinone imine (NAPQI) [63]. Acetaminophen-induced liver toxicity with the concomitant use of phenytoin or phenobarbital or with the use of tyrosine kinase inhibitors was reported in the literature [64, 65]. The interaction is assumed to be due to competition or inhibition of UGT activities. A recent study [66] revealed that the coadministration of a cannabidiol-rich cannabis extract and acetaminophen results in alterations in the livers of aged female mice. As cannabinoids can inhibit UGTs, a higher concentration of acetaminophen might be expected. When glucuronidation is compromised, acetaminophen is directed towards the formation of the reactive metabolite NAPQI resulting in liver damage.

Moreover, acetaminophen is a MRP2 substrate so deregulation of this transporter by the concomitant use of cannabinoids can result in higher levels of the drug as well [67].

### 3.3. Cannabinoids-Antidepressants

Mixed-action antidepressants (serotonin and norepinephrine-reuptake inhibitors) such as duloxetine, amitriptyline, and venlafaxine are a mainstay in the treatment of many chronic pain conditions [68].

Elimination of duloxetine is mainly through hepatic metabolism involving CYP1A2 and to a lesser extent CYP2D6. There is evidence that coadministration of duloxetine with CYP1A2 and CYP2D6 inhibitors increased duloxetine levels [69]. As stated before, CBD is an inhibitor of CYP1A2 and THC, CBD, and CBN inhibit CYP2D6, so if cannabinoids are used as a concomitant medication, an increase in duloxetine plasma levels may be seen.

Venlafaxine relies on CYP2D6 for conversion to *O-*desmethylvenlafaxine (major active metabolite). Further conversion of this metabolite involves CYP2C19 and CYP3A4. Venlafaxine is also metabolized by CYP2C19, CYP2C9, and CYP3A4 but to a lesser extent [70]. As all the enzymes implied in venlafaxine and its active metabolite biotransformation are inhibited by cannabinoids, the clinical implication is difficult to predict. Several studies evaluating CYP2D6 polymorphism [71–73] concluded that higher venlafaxine and lower *O*-desmethylvenlafaxine levels in poor metabolizers resulted in a reduced clinical response with an increased risk for side effects in comparison with extensive metabolizers. Polymorphisms in the CYP2C19 genes that result in decreased enzymatic activity have also been documented [74, 75]. Therefore, elevated venlafaxine levels caused by the potential inhibition of cannabinoids of its metabolic pathway can affect drug response and its side-effect profile.

Amitriptyline is metabolized mostly by CYP2D6, CYP3A4, and CYP2C19, the latter leading to the formation of nortriptyline (active metabolite). Other isozymes involved in amitriptyline metabolism are CYP1A2 and CYP2C9. Based on dosing recommendations made by the Clinical Pharmacogenetics Implementation Consortium in 2016 according to CYP2D6 and/or CYP2C19 variants of individuals [76], if the level of amitriptyline and its active metabolites are too high as happening in poor metabolizers, there is an increased risk of toxicity. Certain drugs as cannabinoids inhibit the activity of these isoenzymes and make normal metabolizers resemble poor metabolizers.

Regarding efflux transporters in the brain, recent studies supported a low possibility that Pgp affects these drugs [77].

To sum up, drug interactions between cannabinoids and antidepressants, if they occur, may be due to metabolizing enzyme inhibition. This inhibition may increase the levels of the antidepressants or their active metabolites resulting in side effects such as the serotonin syndrome, hyponatraemia [78–80], hemorrhagic events [81–84], and QT interval prolongation among others [85, 86]. In the case of duloxetine and amitriptyline, as both drugs are metabolized by CYP1A2, chronic smoked cannabis use may result in lower concentrations of these drugs and perhaps lower efficacy.

### 3.4. Cannabinoids-Anticonvulsants

Antiepileptic drugs are used worldwide to treat several disorders other than epilepsy, such as neuropathic pain, migraine, and bipolar disorder [87]. The first-line options for the treatment of various neuropathic pain conditions are carbamazepine, gabapentin, and pregabalin [88].

Pregabalin and gabapentin share a similar mechanism of action, and both undergo renal excretion [89]. Based on the renal elimination of these drugs, no DDIs between these gabapentinoids and cannabinoids should be expected. With regard to efflux transporters, some authors' results [90] suggested that a combined treatment of pregabalin with Pgp inhibitors enables the prolongation of dose interval of this drug. However, no studies in literature found increased pregabalin levels in the brain with the use of Pgp inhibitors.

Although Gaston et al. [32] did not find changes in carbamazepine levels when administered with cannabis, they focused the study on CBD as the perpetrator drug and carbamazepine as the substrate but information is lacking about the influence of concomitant carbamazepine on CBD plasma levels. Carbamazepine is a well-known inducer of CYP3A4 [91], and therefore, THC, CBD, and CBN metabolism could be affected leading to lower plasma concentrations of these cannabinoids.

Although there is insufficient evidence to support the use of valproic acid for neuropathic pain and fibromyalgia [92], it is sometimes used for these purposes in the clinical practice. Valproic acid is metabolized by three different routes: glucuronidation (UGT1A3, UGT1A4, UGT1A6, UGT1A8, UGT1A9, UGT1A10, UGT2B7, and UGT2B15) and *β*-oxidation (using carnitine as a carrier) in the mitochondria (major pathways) and a minor route (*ω*-oxidation) leading the latter to the formation of a hepatotoxic metabolite (4-en-VPA) [93, 94]. According to some studies [95], valproic acid inhibited UGT1A9 in an uncompetitive manner and UGT2B7 competitively. Glucuronidation is also involved in CBD metabolism being CBD an inhibitor of UGT1A9 and UGT2B7. On the one hand, if cannabinoid concentrations are high, perhaps CBD may impair valproic acid glucuronidation, and thus, valproic acid clearance may be reduced. The higher concentrations of valproic acid induce carnitine depletion [96], and this could increase the *ω*-oxidation route leading to a higher concentration of 4-en-VPA (hepatotoxic metabolite). This last fact could result in incorrect ammonium elimination and thus hyperammonemia [97–99]. On the other hand, valproic acid inhibits UGT1A9 and UGT2B7, both involved in cannabinoid elimination. Perhaps, this inhibition plays the main role and higher concentrations of cannabinoids could be seen in turn. This fact could be supported by the observation made by Gaston et al. [32]. Although these researchers did not measure CBD levels, they did not find a significant change in the valproate levels with increasing doses of CBD but a rise in aspartate transaminase (AST) and/or alanine transaminase (ALT) levels after CBD treatment. These authors concluded that CBD enhances the negative effects of valproic acid on liver functions, but perhaps, valproic acid is the one that intensifies CBD hepatotoxicity augmenting its blood levels. Research done in mice [100] showed that CBD treatment increases liver-to-body weight, ALT, AST, and total bilirubin. In clinical trials carried out recently, some authors [101–103] found elevated liver enzymes in 5-20% of patients treated with CBD, and some patients had to be withdrawn from the studies due to serious hepatic complications. So the combination of CBD with other drugs that exhibit hepatotoxicity and interact with CBD should be of great concern. Valproic acid is not a substrate of Pgp or MRPs [104], so interactions with cannabinoids at this level are unlikely.

Regarding lamotrigine, although the evidence of its efficacy in chronic pain is unconvincing, it can have some effect in patients with painful HIV-related neuropathy [105] and in the prevention of migraine with aura [106]. Lamotrigine is predominantly metabolized by glucuronidation (UGT1A4 and UGT2B7), and it also undergoes elimination by a minor elimination pathway that involves CYP450 enzymes [107]. This minor route converts the drug to a reactive arene oxide metabolite [108]. Such intermediate metabolite, if not effectively detoxified, can result in cellular damage [109]. Skin injuries, Stevens-Johnson syndrome, and toxic epidermal necrolysis are all reported adverse events related to lamotrigine use [110], mainly when the drug is coadministered with valproic acid [111–113], a well-known inhibitor of the glucuronidation pathway. Cannabinoids can act inhibiting UGTs [114] in the same way valproic acid does. So, in the absence of the major pathway, lamotrigine can be bioactivated to the arene oxide and an increased risk of skin reactions in patients could be expected. In addition, lamotrigine is a substrate of Pgp and BCRP [115], so downregulation of their expression provoked by cannabinoids can intensify the drug effect.

A comprehensive overview of the potential interactions discussed in the text is summarized in Table 2.

## 4. Conclusion

Data on significant DDIs between cannabinoids and other medications is still limited and most of it comes from *in vitro* and animal studies. The results obtained in the literature may be of help, but they cannot be extrapolated to human beings. Given that the widespread use of cannabinoids will certainly continue, further research in humans is essential to clarify DDIs in order to fully understand their relevance in the clinical setting.

## Figures and Tables

**Table 1:** Effect of cannabinoids on CYP450 isoenzymes, UGTs, and efflux transporters.

Cannabinoids	CYP P450 isoenzymes	UGTs	Modulation of efflux transporter expression
CBD	Inhibition of CYP1A1, CYP1A2, CYP2C9, CYP2C19, CYP2B6, CYP3A4, and CYP2D6	Inhibition of UGT1A9 and 2B7	Deregulation of Pgp, BCRP, and MRP1-4 transporter expression
THC	Inhibition of CYP3A4, CYP2D6, and CYP2C9	THC-OH, THC-COOH (metabolites) can compete with glucuronidation pathways	
CBN	Inhibition of CYP3A4, CYP2D6, and CYP2C9	Inhibition of UGT1A7, 1A8, and 1A9	

BCRP: breast cancer resistance proteins; CBD: cannabidiol; CBN: cannabinol; CYP: cytochrome; MRP: multidrug resistance proteins; Pgp: glycoprotein P; THC: *Δ*^9^-tetrahydrocannabinol; THC-OH: 11-hydroxy-tetrahydrocannabinol; THC-COOH: 11-nor-9-carboxy-tetrahydrocannabinol; UGTs: UDP glucuronosyltransferases.

**Table 2:** Drugs commonly used in chronic pain, their main metabolic pathways, efflux transporter implication, and the result of potential interaction with cannabinoids.

Drugs	Efflux transporter substrate	Metabolic pathway	Potential cannabinoid interaction
Morphine	Yes	UGT2B7	Augmented analgesic potency due to efflux transporters downregulation. Dose reduction may be required.
Codeine	No	CYP2D6	Possible augmented analgesia provoked by the active metabolite (morphine) by downregulation of efflux transporter expression. Dose reduction may be required.
Oxycodone	Yes	CYP3A4/5, CYP2D6, UGT2B7, and UGT2B4	Augmented analgesia due to parent drug or active metabolite by efflux transporter downregulation and/or enzyme inhibition. Dose reduction may be required.
Methadone	Yes	CYP3A4, CYP2B6, CYP2C19, CYP2C9, CYP2C8, and CYP2D6	Augmented analgesia due to enzyme inhibition and/or efflux transporter downregulation. Dose reduction may be required.
Tramadol	No	CYP2D6, CYP2B6, and CYP3A	Possible augmented analgesia due to inhibition of metabolism of active metabolite. Dose reduction may be required.
Fentanyl	Yes	CYP3A4	Possible augmented analgesia due to inhibition of metabolism and/or efflux transporter downregulation.
Acetaminophen	Yes	UGT1A1, UGT1A6, UGT1A9, and UGT2B15	Higher levels of acetaminophen due to UGT inhibition and/or efflux transporter downregulation and thus possible hepatotoxicity. Monitor adverse effects.
Duloxetine	No	CYP1A2, CYP2D6	Higher concentration of antidepressant due to metabolizing enzyme inhibition. Dose reduction may be required. Smoked cannabis may increase clearance of duloxetine. Monitor for loss of efficacy with chronic marijuana use.
Venlafaxine	No	CYP2D6, CYP2C19, CYP2C9, and CYP3A4	Higher concentration of antidepressant due to metabolizing enzyme inhibition. Dose reduction may be required.
Amitriptyline	No	CYP2D6, CYP3A4, CYP2C19, CYP1A2, and CYP2C9	Higher concentration of parent drug and/or active metabolites due to metabolizing enzyme inhibition. Dose reduction may be required. Smoked cannabis may increase clearance of amitriptyline. Monitor for loss of efficacy with chronic marijuana use.
Valproic acid	No	UGT1A3, A4, A6, A8, A9, A10, UGT2B7, UGT2B15, and *β*-oxidation in the mitochondria (using carnitine as carrier)	Possible higher levels of valproic acid by inhibition of UGTs or higher levels of cannabinoids due to valproic acid UGT inhibition. In both cases, the interaction could result in hepatic damage. Monitor adverse effects.
Lamotrigine	Yes	UGT1A4, UGT2B7	Higher levels of lamotrigine by UGT inhibition and/or downregulation of efflux transporters. Possible cutaneous reactions. Dose reduction may be required.
